# Concurrent Esophageal Dysplasia and Leiomyoma

**DOI:** 10.1155/2014/804175

**Published:** 2014-06-29

**Authors:** Asim Shuja, Khalid A. Alkimawi

**Affiliations:** ^1^Department of Medicine, St. Elizabeth's Medical Center, Brighton, MA 02135, USA; ^2^Department Gastroenterology, Tufts Medical Center, 800 Washington Street Boston, MA 02111, USA

## Abstract

Esophageal leiomyomas (ELMs) are rare but described in the literature. They are usually benign and do not require resection unless they are large and symptomatic. Most of such masses arise from the muscularis mucosa. It is very uncommon to find epithelial dysplasia overlying a subepithelial leiomyoma. A review of the literature reveals only one prior case of ELM with an overlying epithelia dysplasia and here we report a second case.

## 1. Introduction

Leiomyoma (LM) is the most common benign esophageal tumor [1]. It is usually small, asymptomatic, and slow growing. Studies like esophagogastroduodenoscopy (EGD), barium swallow, computed tomography (CT) of the chest, and endoscopic ultrasound (EUS) may aid in diagnosis. Biopsies should not be obtained if LMs are covered by endoscopically normal mucosa, as this may interfere with surgical removal as well as the fact that they have negligible malignant transformation. Surgery is indicated if lesions are large and symptomatic in the form of dysphagia. These tumors are positive for desmin and smooth muscle actin (SMA) stains [2]. It is important to differentiate LM from esophageal gastrointestinal stromal tumor (GIST) because of higher malignancy potential of the latter. LM with overlying squamous cell carcinoma has been reported, but a subepithelial lesion (i.e., LM) with epithelial dysplasia is extremely rare. From our literature review, we report a second case of such kind [3].

## 2. Case Report

**Figure 1:**
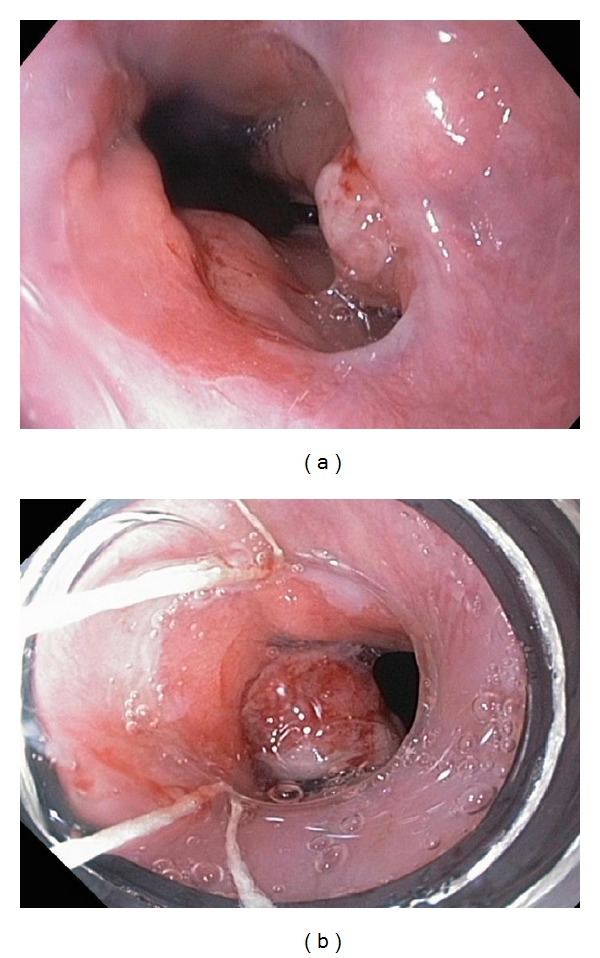
(a) Esophagus: a tongue of Barrett's was found in the distal third of the esophagus. (b): A nodule was found in the GE junction. A mucosal resection (using a Duette EMR kit) was performed, with success.

**Figure 2:**
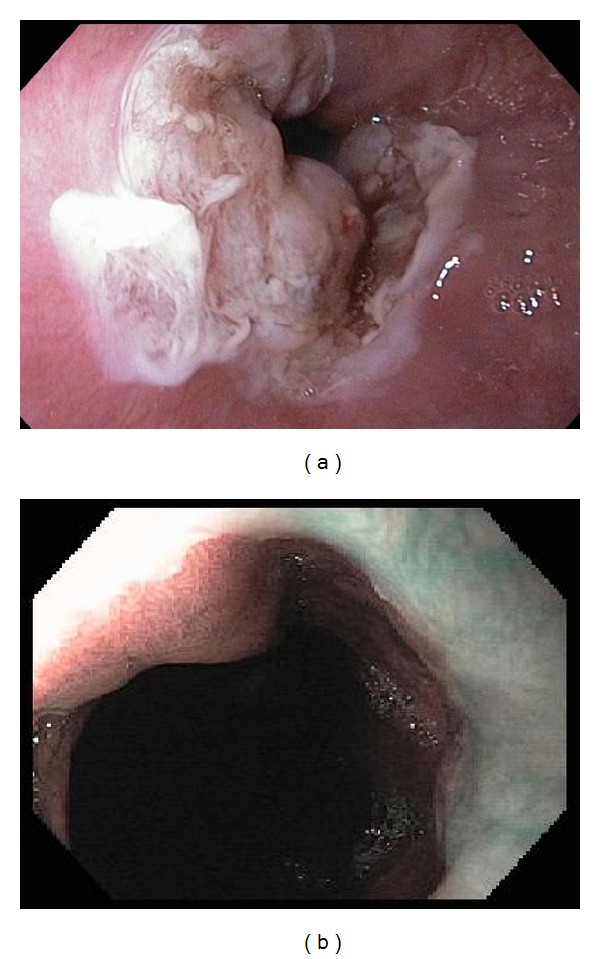
(a) Scar was found in the area of previous mucosal resection. Ablation of Barrett's mucosa was performed with multipolar electrocoagulation with success. (b) A tongue of columnar appearing mucosa was found in the esophagus spanning 1 cm: no residual columnar appearing mucosa.

**Figure 3:**
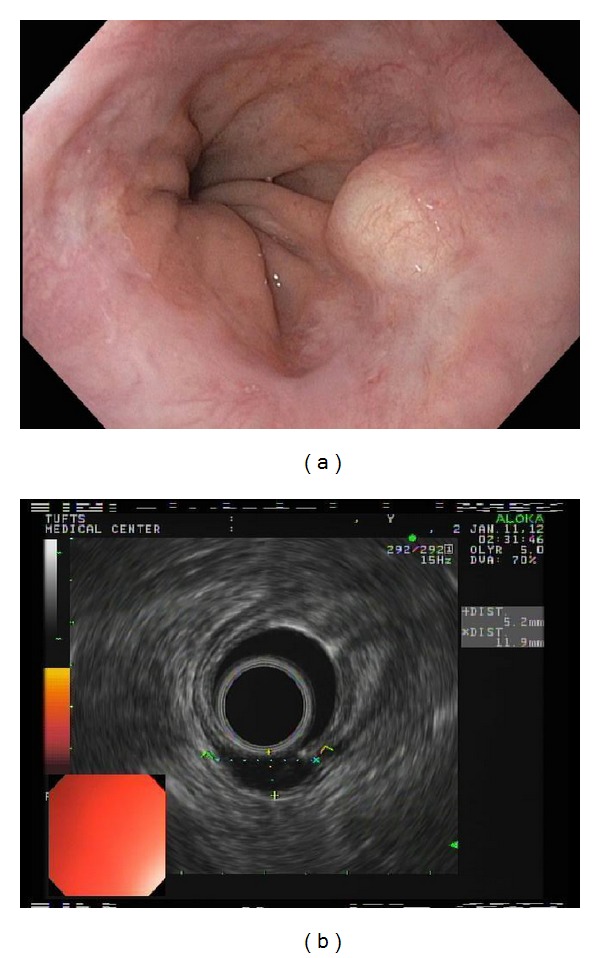
(a) Reappearance of submucosal nodule at the GE junction. (b) EUS: 11 mm × 5 mm nodule arising from the mucosa.

**Figure 4:**
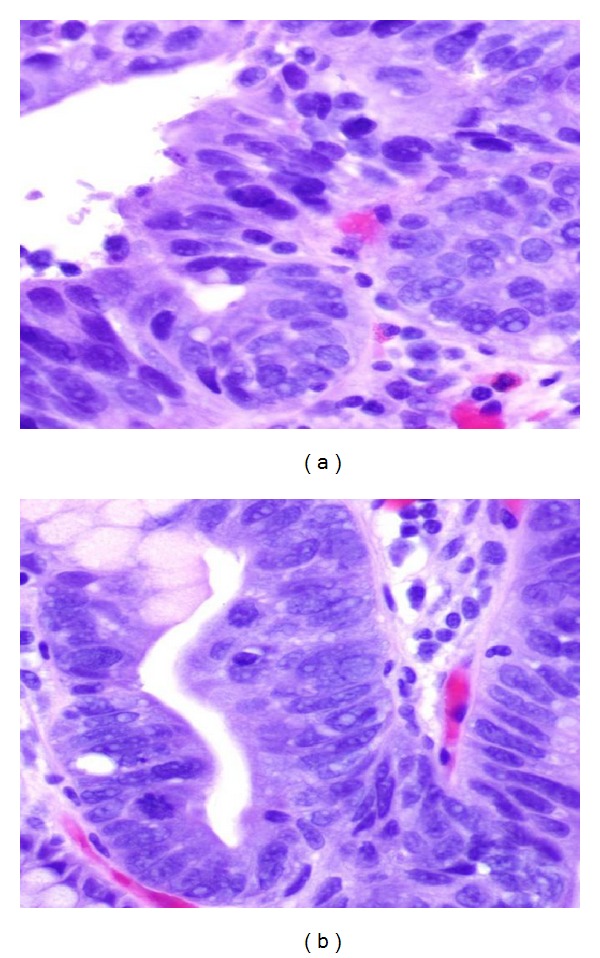
(a) GE junction mucosa with focal intestinal metaplasia and extensive high grade dysplasia/adenocarcinoma* in situ*. (b) High grade dysplasia extends to one margin and detached fragments of high grade dysplasia are also present.

**Figure 5:**
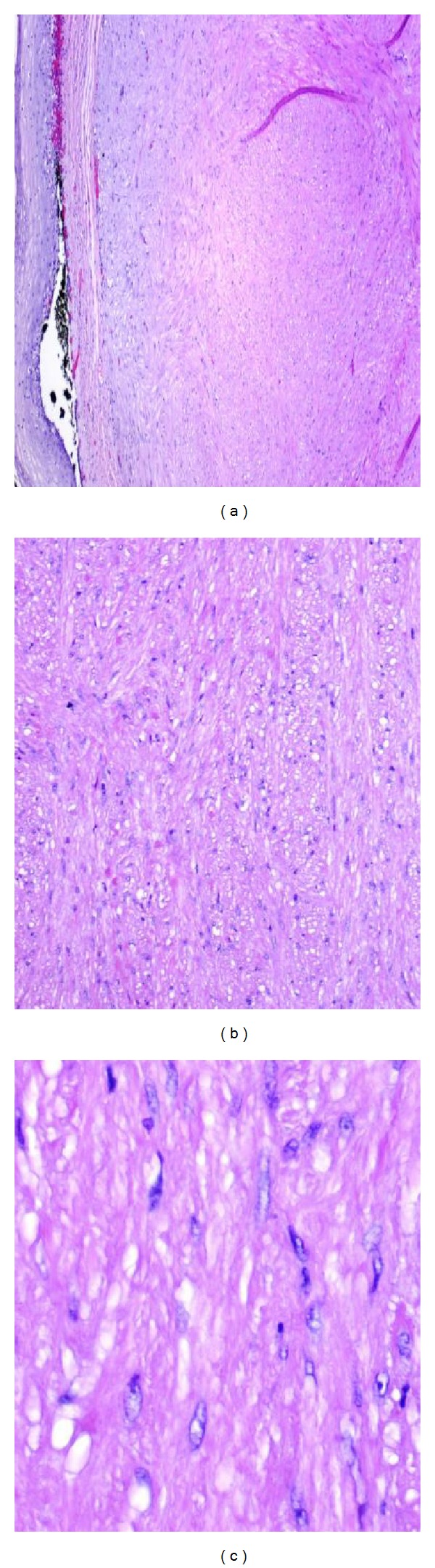
(a) Esophageal LM arising from muscularis mucosa. (b, c) ELM is composed of intersecting bland spindle cells with no mitosis or nuclear atypia.

**Figure 6:**
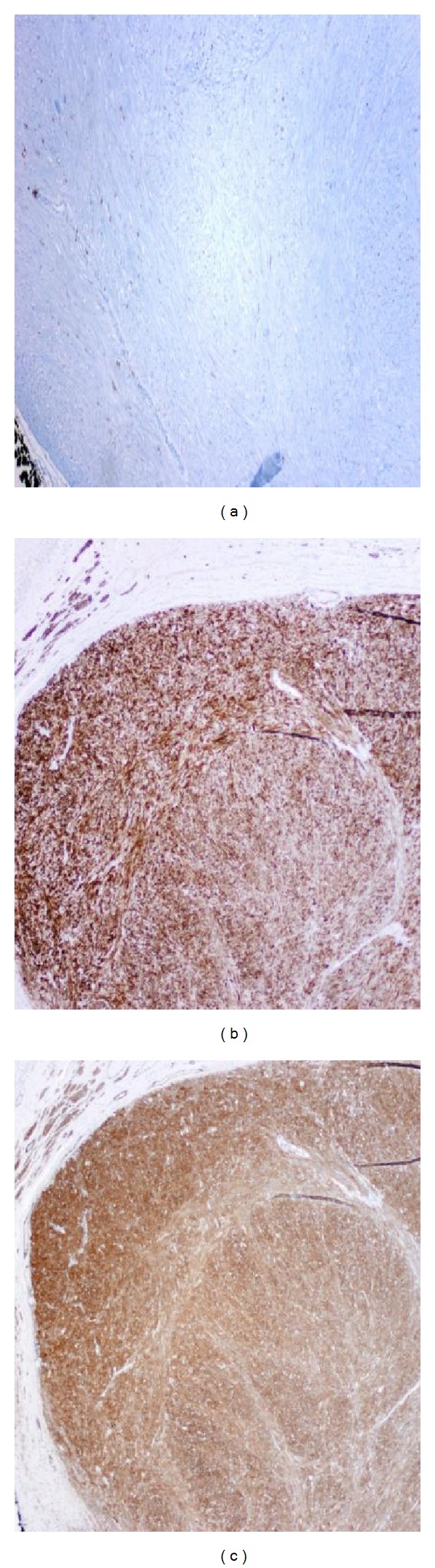
(a) ELM negative for CD117 (C-Kit). (b, c) LM diffusely and strongly positive for desmin and SMA stain, respectively.

**Figure 7:**
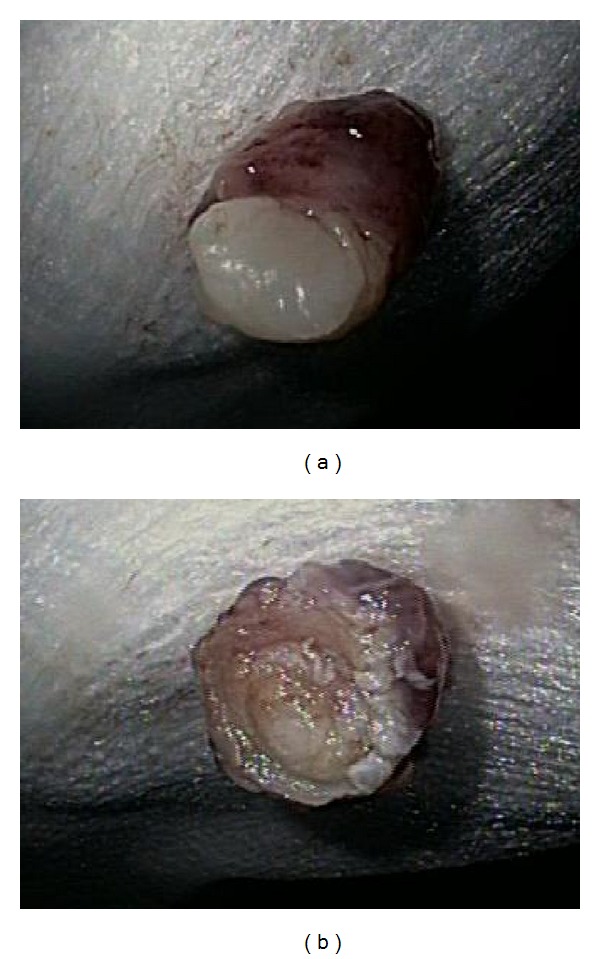
(a, b) 8 mm firm nodule in the distal third/GE junction. It was removed via endoscopic mucosal resection.

A 72-year-old male with history of GERD and Barrett's esophagus presented with an incidental finding of a subepithelial nodule in the gastroesophageal (GE) junction, found on surveillance EGD (Figures 1(a) and 1(b)). GE junction biopsy revealed intramucosal adenocarcinoma and high grade dysplasia, without lymphangioplastic invasion. An endoscopic mucosal resection (EMR) was done and successful ablation of the Barrett's mucosa was performed using multipolar electrocoagulation. The subsequent endoscopy showed normal mucosa and biopsies were negative for any pathology (Figures 2(a) and 2(b)). Three months later, a repeated EGD showed reappearance of a small nodule at the GE junction at the site of previous EMR (Figure 3(a)). This time, the mucosal biopsy was positive for intestinal metaplasia and severe high grade dysplasia. EUS was performed in order to determine the depth and nature of the nodule. It showed an 11.0 mm × 5.0 mm oval, homogenous, hypoechoic mass arising from the mucosa (Figure 3(b)). Fine-needle aspiration biopsy was negative for malignancy. Repeated EMR was performed only for the nodule to recur after few months again, with histological examination of GE junction again revealing high grade dysplasia and adenocarcinoma* in situ* (Figures 4(a) and 4(b)). Histopathology of the esophageal nodule showed superficial fragments of dysplastic mucosa, with spindle cells and no mitotic activity (Figures 5(a), 5(b), and 5(c)). Immunohistochemical analysis revealed cells negative for CD117 (C-Kit) and positive for desmin and SMA (Figures 6(a), 6(b), and 6(c)). A diagnosis of severe dysplasia overlying a small LM from the muscularis mucosa was made. An endoscopic* en bloc* resection was done for removal of the lesion (Figures 7(a) and 7(b)). No procedural complications were observed.

## 3. Discussion

ELM was first well described by von Rahden et al. in 2004 [1]. Although it is the most common benign intramural tumor of the esophagus, it is still very rare, with an incidence of 0.006 to 0.1% on autopsy series data [2]. It is 50 times less common than esophageal carcinoma [4]. ELMs account for approximately 12% of all GI leiomyomas [5]. It is typically found in the 20–60-year-age group with male preponderance (M 2 : 1 F) [6]. Lower third of the esophagus (50%) is the most common site of involvement, followed by the middle third (40%) and upper third (10%), which is consistent with the normal anatomical distribution of smooth muscle within the esophageal wall [7]. The size of the LMs can range from less than 0.5 cm (microleiomyomas) to as large as 30 cm. These tumors rarely cause symptoms when they are smaller than 5 cm in diameter. Large tumors can cause dysphagia, vague retrosternal discomfort, and so forth [2]. In extreme cases where severe esophageal obstruction is caused by a LM, weight loss and muscle wasting may be observed [8].

EGD, EUS, CT of chest, and so forth may aid in diagnosis. On endoscopy, LM is identified as a submucosal, freely movable mass with intact mucosa [9]. EUS finding of leiomyoma, homogenous, and hypoechoic mass is the key for differentiating leiomyoma from invasive cancer [10]. LMs may occur in the muscularis propria layer but is most common in the muscularis mucosa of the esophagus [11]. If a LM is suspected and the overlying mucosa is normal, biopsy should be avoided, for it is likely to be nondiagnostic and may increase intraoperative complications [12]. Microscopically, they have low cellularity and are composed of interlacing fascicles of bland spindle-shaped smooth muscle cells. There is minimal nuclear atypia and no rare mitotic activity seen. The characteristic morphology makes the histological diagnosis of esophageal LM relatively easy, and usually no immunohistologic studies (IHS) are required. However, in cases where there is moderate cellularity with some nuclear atypia, other tumors such as GIST and schwannoma are included in the differential diagnosis. IHS for SMA and desmin show positive staining in LM and they are negative for CD117 (C-Kit), CD34, or S100. Although GIST of the esophagus is even rarer, it is important to differentiate it from LM, because esophageal GIST is a more aggressive tumor [11]. In contrast to LM, GISTs are positive for CD117 and CD34 [12–14]. Malignant transformation is extremely rare. In review of 800 cases in the world literature, only two (0.2%) cases were documented to show malignant transformation [2].

Treatment for ELMs depends on multiple factors, including tumor size, location, appearance, and the patient's symptoms and overall conditions [15, 16]. Asymptomatic patients can be monitored by endoscopy and radiology [17]. The indications for surgical treatment include unremitting symptoms, progressive increase in tumor size, mucosal ulceration, the need to obtain histological diagnosis, and facilitation of other esophageal procedures [11]. Recurrence of the ELM is extremely rare [18].

The coexistence of an epithelial lesion and a subepithelial lesion is rare. To our knowledge, twelve patients in ten case reports with carcinoma located in the mucosa overlying a benign tumor have been reported [19–21]. All of these cases were squamous cell carcinoma. However, from our experience, this is the second case of epithelial dysplasia overlying a LM in the esophagus. It is difficult to understand whether these dysplastic changes were related to his underlying LM or they were a precursor to his Barrett's. Nodule was removed via EMR, and surveillance EGD performed 6 months later did not show recurrence of the mesenchymal tumor nor showed any dysplastic changes in the mucosa. We speculate that the coincidence of LM and epithelial dysplasia is a very rare finding which needs proper surveillance.

## 4. Conclusions

In conclusion, leiomyomas of the esophagus can rarely be found underneath a severely dysplastic mucosa. Endoscopic removal is a suitable option for lesions arising from muscularis mucosa, if detected early.
